# Supplementary material for: Segmenting white matter hyperintensities on isotropic three-dimensional Fluid Attenuated Inversion Recovery magnetic resonance images: Assessing deep learning tools on a Norwegian imaging database
Source: PLoS One. 2023 Aug 24;18(8):e0285683. doi: 10.1371/journal.pone.0285683 (PMC10449185; doi:10.1371/journal.pone.0285683)
Supplement: Attachment — Submitted filename: PLOSOne_R2V2_responsedoc.docx.pdf [file pone.0285683.s002.pdf]

Dear Niels Bergsland, Academic Editor, PLOS ONE, and Reviewers

Regarding PONE-D-22-24185

We are pleased to submit a revised manuscript. Reviewer-1 stated: "*All comments have been addressed*". Reviewer-2 had some additional comments that were quite helpful to improve the overall clarity of this manuscript.

We denote reviewer comments in **black** font and our "**Authors reply**" using blue font.

We are grateful that PLOS ONE is interested in this research.

On behalf of all co-authors,
Signed sincerely,
Martin Soria Røvang

Responses to Reviewer 1:
No responses were requested. We thank Reviewer 1.

Responses to Reviewer #2:

I would like to recognize and thank the authors regarding the work performed in order to address some of the concerns raised during this first exchange. Notably, precision concerning the different kinds of acquisitions, what was performed in-house and not, the concision effort on some parts of the article and the development of aspects that required additional explanations are very welcome.

Nevertheless, despite these positive improvements, this new version raises a lot of new interrogations, that I would consider at least as important as the ones discussed during the first round.

The objective of the article, from my understanding, is to report work performed in order to provide an automated WMH segmentation from 3D FLAIR images to clinicians. Work has been done and is available in the literature on this topic, but presents differences with the one reported here. Deep learning-based methods handling this segmentation task have been proposed, but based on 2 images, 3D-T1 (2D T1 is still mentioned instead of 3D in the introduction) and 2D FLAIR. Other methods use 3D FLAIR, but are not based on deep learning methods.

R2.1 Authors reply: We appreciate the summary provided here and feedback that indicates the revised manuscript was an improvement. Regarding "2D T1", we fixed this typo.

I disagree with the authors regarding their response to reviewer 1 (R1.8), as the main objective of this work being to segment WMH from 3D FLAIR data, the other methods related to this application should be compared to the proposed models. Indeed, despite the fact that DL-based methods have been shown to provide increased performance metrics on a number of applications compared to previous works, the "superiority" of the DL-based models needs to be proven against gaussian-mixture models (GMM) or the method proposed in Zhong et al, 2014 for this particular case. Moreover, the authors

mention the use of GMM as part of their semi-automated annotation procedure, which I understand as the authors having effectively used this method, which would have then been easily compared. This comparison would have been, in my opinion, at least as meaningful as the comparison with a 2 input images model, from which we could have expected the lower performance metrics, due to the absence of this bayesian model retraining.

R2.2 Authors reply: Thank you for the comment. And some clarification is needed. The ground truth in the current study did indeed come from the result of the GMM; this approach was iterative and executed by a clinician with domain expertise to arrive at a WMH segmentation that was deemed adequate as a ground truth.

The methods section describes that the GMM model will have been run several times if the experts did not approve the initial estimate. The reason for this choice of GT was that no DL-based method was readily available at the time of analysis. Further, a fully manual segmentation was not feasible, given the amount of volumetric data to be annotated. Using an established and available non-DL method was therefore chosen as a suitable alternative. Therefore, we cannot show superiority over the GMM since it served as the ground truth.

Although we could have compared our model to other non DL-based models, we feel that this is somewhat outside the scope of the current study. We emphasize that DL-based analysis has the advantage of being more conducive to retraining a model (i.e. as new data becomes available). We have tried to make it even clearer in the discussion that the ground truth is derived from a GMM method and why we have chosen to compare against the other DL-based models.

The medical images processing community is very familiar with the Unet architecture and the nnUNet framework, but not with the models presented here as state of the art (Hypermapp3r) or the 2.5-D Unet. In particular, the selection of the 2.5-D model should be more motivated, and the choices made to generate it really detailed in the publication, as the reference provided to the readers for explanations is the main author's master thesis report.

R2.3 Authors reply: The rationale for including a 2.5-D model was based on the literature that suggests that 2D models outperforms 3D models, as was the case for the WMH data challenge. Initial results (as referred to in a Master's thesis) also indicated that the 2.5-D model, combined with multi-planar analysis, incorporating the staple methods - performed well for WMH segmentation and hence was a natural starting point for comparison to 3D models. We added this in the discussion. We also expanded slightly on the Monte Carlo dropout in the network architecture.

In the results and discussion sections, the nnUNet model is presented as the one providing the overall best performance metrics. From the clinical point of view, is the coverage of the large lesions of the highest importance, or the capability of a model to actually detect lesions, even the smaller ones ? If detecting lesions has a significant meaning for clinicians, is the selection of the nnUNet model still relevant ? For an example of lesions detection evaluation, an interesting reference could be Commowick et al., 2018 (doi: 10.1038/s41598-018-31911-7)

R2.4 Authors reply: This is a good point; the 2.5-D model seems to perform better for smaller lesions in the scatter plot; however, this model also tends to generate more false positives (i.e. segments too

many locations), which is undesirable. On balance, we believe that the 3D nnUNet framework provided the best results across the range of cases considered.

The Hypermapp3r model is presented as having some appreciable advantages. The lower number of parameters, compared to the 2 other models, would be an advantage regarding model's deployment, and this bayesian model can provide uncertainty related information. But the perspective of retraining / fine-tuning this model or adapting a model with these properties to the 3D-FLAIR WMH application is not envisaged.

R2.5 Authors reply: Indeed the Hypermapper has favorable features, such as the Monte Carlo dropout functionality. We have now added a short section in the discussion on how features from the different models could be combined to give the same favorable prediction results. The Hypermapper model did have fewer parameters, but since it had to predict 20 times and required skullstripping, it had a longer prediction time than the other two models.

The authors did not provide information about the reason of the population description changes (number of subjects modified from 642 to 441, that influences all the related subsets).

R2.6 Authors reply: We apologize for this confusion. We attempted to clarify this in the previous revision. Indeed the total sample size is N=441 and this corresponds to the number of unique participants. We opted to remove the repeat MRI sessions that were available for some participants. This "extra" data was not needed to convey the main objectives of this study. And all the results are now updated with the revised sample size.

Overall, despite interesting aspects presented in this paper, I understand it as a compilation of valuable work, that unfortunately lacks investigations and does not allow to construct and respond to the story that could be attached to the main objective.

R2.7 Authors reply: We appreciate this feedback. The revised manuscript now has additional statements in the Introduction. For convenience, text is pasted here:
"*Prior WMH segmentation research demonstrates the feasibility of segmentation based on has used 3D FLAIR images [11,12]; there is a need to extend this research with fast and automated methods of which deep learning based approaches are highly relevant and enable to large sample size cohorts. Therefore the primary objective of this work is to examine deep learning solutions that can yield robust segmentations across field strength and site.*"
And the revised hypothesis is now:
"*We hypothesized that the two in-house trained models that used 3D FLAIR-weighted images data would achieve high WMH segmentation performance using a battery of widely accepted performance metrics for internal and external data sources.*"
