# Supplementary material for: Segmenting white matter hyperintensities on isotropic three-dimensional Fluid Attenuated Inversion Recovery magnetic resonance images: Assessing deep learning tools on a Norwegian imaging database
Source: PLoS One. 2023 Aug 24;18(8):e0285683. doi: 10.1371/journal.pone.0285683 (PMC10449185; doi:10.1371/journal.pone.0285683)
Supplement: Attachment — Submitted filename: response_to_reviwers.docx [file pone.0285683.s001.docx]

Dear Niels Bergsland, Academic Editor, PLOS ONE, and Reviewers

Regarding PONE-D-22-24185

We are pleased to submit a revised manuscript and appreciate the thoughtful comments from two reviewers. We are encouraged that one reviewer stated “*this is an interesting analysis*”.

In this revision, we summarize the major changes:

- An improved description for the study rationale; and how the data reflects research embedded in clinical neurology services in Norway.
- In some cases we noted that the same participant was used in validation and test sets (by virtue of the repeat imaging sessions); we opted to fix this issue, re-train the models, and update the results accordingly (only minor changes occurred for the results).
- We revised all the figures after recognizing similar comments from the reviewers.

We denote reviewer comments in **black** font and our “**Authors reply**” using blue font.

We are grateful that PLOS ONE is interested in this research.

On behalf of all co-authors,

Signed sincerely,

Martin Soria Røvang

**-------------------------------------**

**Responses to Reviewer #1:**

Reviewer #1: The authors investigated the use of deep learning approaches to segment 3D FLAIR data. They collected and used data from a large multisite dementia initiative to test two 3D deep models for segmenting WMH (one trained internally and one without any further training) compared to a Bayesian deep network. The models were trained on a subset of 642 participants and tested on a subset as well as an external dataset. Some subset of data was manually segmented to evaluate and train the model. They found that the 3D nnU-net performed best outperforming other models with some caveats. This is an interesting analysis, however there are some issues that should be addressed:

1. In the 2.6.1 section, many of these decisions are described but not critically reasoned. It would be helpful to justify the reasoning behind various parameter choices in the manuscript (even if they are just based on prior experience and work).

**R1.1 Authors reply:** We added reasoning for the parameters. In short, parameters were chosen based on values used in cited papers, preliminary findings, and internal testing. An alternative would be to do a full grid search across the parameter space, but this was considered to be too computationally expensive and not feasible with our available compute resources.

2. The performance on the external dataset is quite low in general. The authors should comment on whether intensity normalization approaches may help generalize these types of approaches.

**R1.2 Authors reply:** We first point out that a revised Figure 1 shows the normalized intensity distributions for each of the training sites. We added the following to the limitation section of the Discussion:

*“We observed a ~12% drop in DSC on the external test data. One possible reason the external dataset had low performance metrics may be due to the low number of Prisma scans available for training.”*

*We have added a statement that histogram matching could potentially improve the performance on the external test set”.*

3. The authors state: “However, since the models tested here were not trained on the MICCAI dataset, a direct comparison of performance may be misleading.” The MICCAI is an open challenge, did the authors consider submitting results to identify how well they performed in that dataset? Otherwise, if this is not important then these types of statements should be removed.

**R1.3 Authors reply:** This would be interesting to do, but we have removed any comparison to this challenge.

4. The authors state: “Our results do, however, suggest that T1-weighted images may not be needed for WMH segmentation.” Why is this important especially given that a T1-weighted image is almost always available if a FLAIR is available?

**R1.4 Authors reply:** We thank the reviewer for this point as it is an opportunity to clarify that our intention is to deploy the WMH segmentation tool in clinical settings, therefore a single image input is highly desirable.We added the following statement to the Introduction to support this:

“*Exclusive requirement of a FLAIR acquisition is desirable during clinical deployment or batch production of WMH segmentation.”*

5. The conclusions of the manuscript leave a lot to be desired. While the initial motivation for the paper seems relevant, the conclusions are quite flat. For instance, they state that models can be trained on 3D imaging data, it is unclear why this is important to establish. This seems like something that should be feasible. They also state that a T1-weighted image would not be necessary, but this (see previous comment) is also not that important given that they are often available (unless there is motion).

**R1.5 Authors reply:** We appreciate the comments, but we still believe this to be an important point due to the fact that 2D FLAIR is still the standard in many institutions, and therefore most current models are trained on 2D multi-slice FLAIR data. In Norway, however, the standard FLAIR protocol is isotropic volumetric 3D, and it is therefore of major importance to develop a WMH segmentation model with optimal performance in 3D data for use in our national multicenter dementia research initiatives. Regarding the inclusion of T1-weighted, we refer to our reply to Item 4 above. Internal testing and external models shows that adding T1 does not give sufficient enough increase in score to be worth it.

6. The authors also should revisit their hypotheses from the introduction explicitly.

**R1.6 Authors reply:** We modified the hypothesis sentence in the Introduction.

7. It is unclear how many manual segmentations were completed. How many slices were manually segmented

**R1.7 Authors reply:**  We attempted to improve the description on the method to create ground truth segmentations and we note that no slices were segmented manually. Here is the revised text that appears in the Methods:
“*One expert was in the loop during a semi-automated method designed to produce iterative WMH segmentation binary masks until the expert deemed the segmentation to be accurate. A second expert was consulted for challenging cases; in these cases, iterations were performed until consensus was reached.*”

8. In the introduction, the authors should include a paragraph on why it is important to develop 3D FLAIR segmentation algorithms. What challenges exist in this space? What has been done previously? Are there any tools that have been previously developed for 3D FLAIR? If so, why are they not used here? Or why do they not work?

**R1.8 Authors reply:** This is important because it is the standard in Norway. The large MICCAI challenge and the Deep bayesian reference model use 2D FLAIR together with 3D T1. Previous methods exclusively based on 3D FLAIR have not been deep learning methods.

Minor

1. The figure resolutions on all images are quite poor. They should all be redone to higher resolution images.

**R1.9 Authors reply:** Will be fixed with a new upload.

2. Tables 3 and 5 show the same results as figures 2 and 3. Just one set can be presented.

**R1.10 Authors reply:**  We now only show boxplots and a lesion size based scatter plot

3. Some results are presented multiple times in the text, tables, and figures. For instance, for the internal test – the authors state: “For the internal test set, average (+/- std.dev) DSC scores were 0.70 (+/- 0.13), 0.78 (+/-0.10), and 0.63 (+/- 0.15), for the 2.5D U-Net, 3D nnU-Net and Deep Bayesian models, respectively.” They then present this in table 5 and figure 3. It would be better to show it, for instance, in one table and then just state the finding: “3D nnU-Net performed best across all metrics in the internal data (see table 5).”

**R1.11 Authors reply:**  Added best model and referenced to boxplots. Kept DSC mean +- std for each model for reference.

**Responses to Reviewer #2:**

Reviewer #2: The authors present an evaluation of 3 models/algorithms for segmentation white matter hyper-intensities (WMH) on 3D T2 FLAIR MRI images. One of the models has been implemented and trained by the authors. The second model is the default nnUnet implementation, and the third one is a deep Bayesian model. Two of these models are trained on the Norwegian Disease Dementia Initiation (DDI) dataset, and tested on a subset of the data associated with this study, as well as on an additional external dataset. The main difference between these two networks and the Bayesian one is that the later was trained on combinations of T2-FLAIR and T1 acquisitions.

The main outcomes are: a demonstration that segmenting WMH on 3D T2-FLAIR acquisitions is feasible, and that the nnUnet gives the best evaluation metrics.

English language skills are globally satisfactory, but the paper structure is sometimes difficult to follow. As an example: there is a pre-processing section, 2.5, but data pre-processing (related with the nnUnet) is not detailled here, rather in a later section, 2.6.2. Inconsistencies across section 2.7 and section 2.8 and the results create additional confusion. While test metrics and statistics are presented in section 2.7, presentation of results start with a not introduced section about WMH volume distribution of the data. In addition, TP, FN and FP are used as a performance indicators, but they are not presented in sections 2.7 and 2.8.

**R2.1 Authors reply:**  We appreciate the opportunity to clean up the manuscript and improve the overall clarity. We moved text according to the reviewer’s suggestions.

In the introduction, the authors assess that so far WMH segmentations have mostly been attempted on 2D FLAIR acquisitions. Could you please provide citations (line 102, line 111 again) ? Nevertheless, one cited paper (Forooshani et al., 2022), is not discussing the use of 2D images. Instead, a 3D Bayesian convolutional neural network is proposed. A confusion between T2-FLAIR and 2D FLAIR may have occurred ?

Moreover, the two articles presented as state-of-the art solutions are citation 5 (citation number 7) and citation 6 (citation number 10): perhaps there is more out there in the literature ? In addition, the Bayesian model cited as SOTA solution requires the use of both T1 and T2 acquisitions, while the objective of the article is to perform segmentation on T2-FLAIR, which creates some confusion.

**R2.2 Authors reply:**  There was some confusion in the original submission, which we have corrected. Namely, Foorshani et al used 3D T1-weighted & 2D T2-weighted FLAIR image inputs into their 3D model. Whereas, we used 3D FLAIR images only, which corresponds to a ~3x higher spatial resolution in the “z-dimension” for the current (Norway) data compared to Foorshani et al.

Some parameters in the equations, as in equation (1), are not explained by the authors (alpha and beta in this case).

**R2.3 Authors reply:**  As above (in **R1.1**), we made this section more succinct and also added better description for the parameters.

The level of details is unbalanced: on one side there is an extensive description of Python libraries for splitting data into train/val subsets, on the other the N4 implementation or DICOM conversion tools are not detailed at all. The latter appears to me as of higher value for reproducibility purpose. Also, details are provided concerning the DDI study, that are of interest, but not of the highest value regarding the work here presented. The reader is being referred to another article to get this valuable information.

Inconsistencies arise between text and tables. Regarding the DDI study, 5 national sites are mentioned, whereas 13 institutions are presented in the corresponding table. The origin or meaning of “institutions” should be explained, as it is difficult for the reader to associate them with the written details. Lines 387-389: three intervals are associated with 4 values, another example of inconsistency.

**R2.4 Authors reply:**  We added N4 and DICOM conversion tools. We also clarified that it was a scanner site and not an institution (i.e. some sites have multiple scanners). We revised the Lines 387-389 in the original submission.

While the first model is presented as the in-house model at the beginning of the article, the nnUnet implementation is also referred as an in-house solution in later sections. This creates confusion: the nnUnet is not a solution designed and implemented by the authors (see line 410 just as an example).

**R2.5 Authors reply:**  We clarified that “in-house” refers to models that were trained de-novo in our laboratory.

The methods are too different to be compared. In fact, they differ in terms of input needs (1 vs 2 contrasts), pre-processing, training and validation splitting (what would have other folds of the nnUnet cross-validation procedure generated as results ?) and prediction (test time augmentation in the case of nnUnet). This level of differences makes it hard to appreciate the cause of different performances. Would some technical choices made in the nnUnet model help the in-house model to learn better on the training set ? I suppose that having at least the same pre-processing and prediction steps would be necessary for sake of comparison.

**R2.6 Authors reply:**  The reviewer makes some interesting points as there is a diversity of approaches. The revised manuscript now addresses performance differences in a more objective manner. We updated the Discussion to include the following on this matter:

“*Several sources could have contributed to differences in performances for the models, i.e. data inputs, image processing steps, and architecture. Therefore, it is important to note differences in performance on a relative but not an absolute scale.*”

An additional concern is associated with the use of the results obtained during the MICCAI WMH segmentation challenge as scores to which the proposed algorithms shall be compared. Nevertheless, comparing results obtained on different data in a single table appears as unsuitable to establish relevant conclusions.

**R2.7 Authors reply:**  Comparisons to MICCAI results have been removed.

Lines 423-428 are unclear. Is the objective of the work to provide a clinically relevant solution, or to obtain the best performance scores ?

**R2.8 Authors reply:**  Indeed the objective of this manuscript is to describe a clinically viable solution. We removed the part that said we focused on larger lesions and now show clear results for all brackets. Objective was to find the model with the best overall score for the five metrics.

In conclusion, the fact that nnUnet performs the segmentation of WMH on 3D T2-FLAIR acquisitions is an interesting result. The comparison with the two other models does not appear relevant because of the level of differences in implementation, inputs requirements and results. The aspect concerning work performed on 2D T2-FLAIR acquisition should be clarified, as 3D acquisitions are a standard.

**R2.9 Authors reply:**  We thank the reviewer for the valuable perspective. We revised text in the Introduction and the Conclusion sections to better articulate the findings. For convenience we paste conclusion text here:
“*Our findings indicate that models relying on single 3D-acquired FLAIR image inputs produced WMH segmentation masks that were comparable and slightly better than a SOTA approach that used pairs of MR images. This research helps to support efforts towards large scale WMH assessments, particularly for our national Norwegian clinical research initiative.*”
